# Supplementary material for: Optical charge injection and coherent control of a quantum-dot spin-qubit emitting at telecom wavelengths
Source: Nat Commun. 2022 Feb 8;13:748. doi: 10.1038/s41467-022-28328-2 (PMC8826386; doi:10.1038/s41467-022-28328-2)
Supplement: Supplementary file 1 — Supplementary Information [file 41467_2022_28328_MOESM1_ESM.pdf]

Supplementary Information: Optical Charge Injection and Coherent Control of a Quantum Dot based Spin-Qubit at Telecom Wavelength

Łukasz Dusanowski,[1,2,*] Cornelius Nawrath,[3] Simone L. Portalupi,[3] Michael Jetter,[3] Tobias Huber,[1] Sebastian Klembt,[1] Peter Michler,[3] and Sven Höfling[1,4]

[1] *Technische Physik and Würzburg-Dresden Cluster of Excellence ct.qmat, University of Würzburg, Physikalisches Institut and Wilhelm-Conrad-Röntgen-Research Center for Complex Material Systems, Am Hubland, D-97074 Würzburg, Germany*

[2] *currently at: Department of Electrical Engineering, Princeton University, Princeton, NJ 08544, USA*

[3] *Institut für Halbleiteroptik und Funktionelle Grenzflächen (IHFG), Center for Integrated Quantum Science and Technology (IQ^{ST}) and SCoPE, University of Stuttgart, D-70569 Stuttgart, Germany*

[4] *SUPA, School of Physics and Astronomy, University of St Andrews, KY16 9SS St Andrews, UK*

Keywords: spin control, hole qubit, quantum dot, telecom C-band

CONTENT DESCRIPTION

In this supplementary information document, we provide further details on the experimental setup scheme (1), non-resonant photoluminescence at B = 0 (2), magneto-photoluminescence (3), and the rate-equation model (4).

SUPPLEMENTARY NOTE 1: EXPERIMENTAL SETUP

For all experiments, the sample is kept in a low-vibration closed-cycle magneto-cryostat (attoDRY2100) at a temperature of ∼1.6 K. A superconducting magnet surrounding the sample chamber is used to apply a magnetic field up to 8 T. The optical part of the setup consists of a home-built confocal microscope mounted on top of the cryostat, and an apochromatic lens with a numerical aperture NA of 0.68. The sample is mounted on piezoelectric stages (Attocube) for control of its position with respect to the lens. Non-resonant excitation of QDs is performed using a pulsed mode-locked Ti:Sa laser (Coherent Mira) at 850 nm and 76 MHz repetition rate (2 ps pulse width). For resonant excitation, two lasers are used: (i) a continuous wave tunable diode laser at 1550-1570 nm (iBlue ModBox) and (ii) a tunable Optical Parametric Oscillator (Coherent OPO-X) pumped by the Ti:Sa laser at 850 nm. The OPO generated optical pulses are shaped spectrally using a fiber-based tunable grating band-pass filter with adjustable bandwidth (EXFO XTM-50). For the Ramsey interference experiments, a laser beam is passed through the free-space Mach-Zender interferometer where the time delay between the pulses is controlled via a retro-reflector mounted on the motorized translation stage. The fluorescence signal is collected through the same lens. A 1200 nm long-pass filter in the confocal microscope filters out the 850 nm laser excitation. Furthermore, polarization optics is set-up in cross-polarization configuration for resonant laser suppression. Additional suppression of the laser is achieved by spatial filtering using single-mode fiber. Emission is then sent to a 75 cm focal length monochromator where it could be resolved spectrally and imaged on the array detector (Princeton Instruments NIRvana) or filtered spectrally and coupled into a superconducting single-photon counting detector (SSPCD, Single Quantum EOS). The SSPCD time resolution is better than 20 ps, the quantum efficiency is over 70%, and the dark count rate is around 100-200 cps. The signal from the single-photon counting detectors is sent to a time-to-digital converter module (Pico Harp 300) triggered by the laser. The total timing resolution of the setup (instrumental response function fwhm) is around 30 ps. The spectroscopic configuration used is shown schematically in Fig. 1.

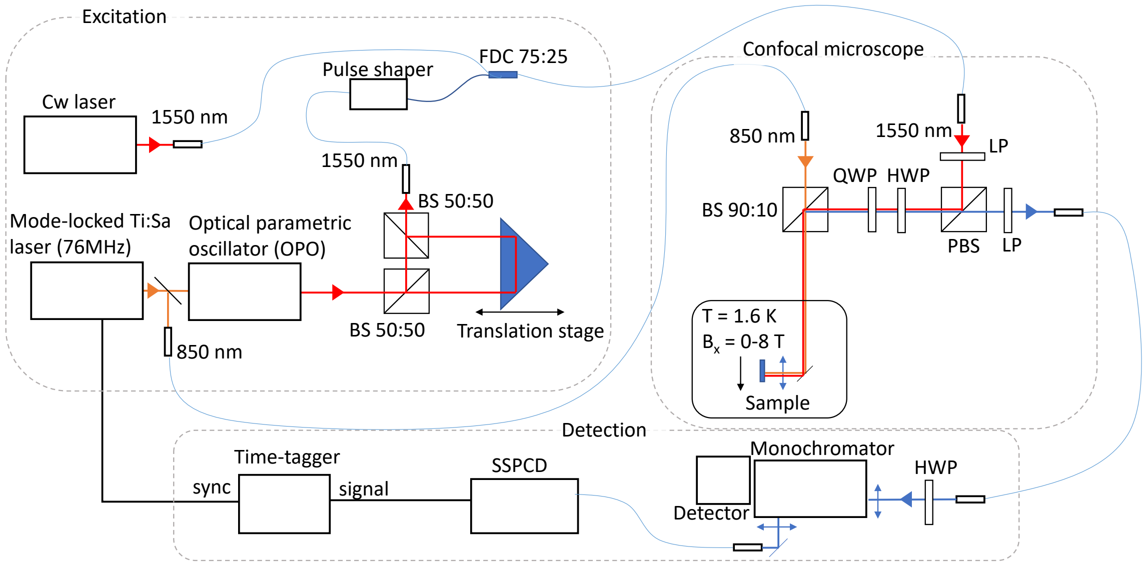

FIG. 1. **Experimental setup scheme.** The sample is kept in a low-vibration closed-cycle magneto-cryostat at a temperature of ∼1.6 K. For optical excitation and PL signal collection, we use an apochromatic lens mounted on a periscope inside the sample chamber. Non-resonant excitation is performed using a pulsed mode-locked Ti:Sa laser at 850 nm and 76 MHz repetition rate (2 ps pulse width). For resonant excitation a continuous-wave tunable diode laser at 1550-1570 nm and a tunable Optical Parametric Oscillator (OPO) pumped by the Ti:Sa laser is used. For the Ramsey interference experiments, a laser beam is passed through a free-space Mach-Zender interferometer, where the time delay is controlled via a retro-reflector mounted on the motorized translation stage. For polarization control, a half-wave plate (HWP) and a quarter-wave plate (QWP) combined with a linear polarizer (LP) are used, set-up in cross-polarization configuration for resonant laser suppression. Emission collected into the single-mode fiber is sent to a 75 cm focal length monochromator where it is resolved spectrally and imaged on the array detector. Alternatively, a signal is filtered spectrally and coupled into a superconducting single-photon counting detector (SSPCD). The electronic signal from the SSPCD is sent to the time-tagger module synchronized with the laser.

SUPPLEMENTARY NOTE 2: NON-RESONANT PHOTOLUMINESCENCE AT B = 0

In Fig. 2 a polarization-resolved zero-field PL spectrum of the investigated quantum dot is shown under non-resonant, pulsed excitation at 850 nm. For that, a half-wave-plate is rotated placed before a fixed linear polarizer located in front of the signal detection fiber (see Fig. 1). The investigated line does not show any polarization splitting within setup resolution and exhibits slight linear polarization with a degree of 11%, which we attribute to the light-hole-heavy-hole bands mixing. More detailed experimental and theoretical studies of excitonic transitions in investigated InAs MMB QDs are summarized in Ref. [1] and indicate a dominant contribution of positively charged complexes in the emission spectra. Following on the above observations and earlier studies we attribute the investigated emission line to a positively charged exciton.

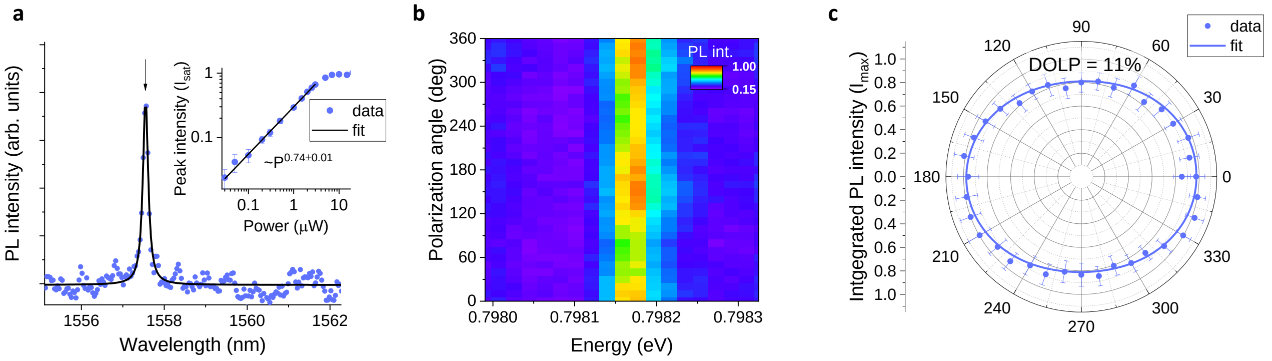

FIG. 2. **Non-resonant photoluminescence.** (a) Low temperature (1.6 K) photoluminescence spectrum of the investigated QD under pulsed 850 nm excitation. The emission line identified as a positive trion is marked with an arrow and fit using Lorentzian function. Inset: PL intensity vs laser pumping power dependence. (b) Color coded PL emission spectra versus detection linear polarization angle. Polarization splitting is not observed for the investigated line within the setup resolution. (c) Polar plot of investigated line emission intensity versus detection linear polarization angle. Line exhibits slight linear polarization with a degree of 11% (most likely due to the light-hole-heavy-hole bands mixing).

SUPPLEMENTARY NOTE 3: MAGNETO-PHOTOLUMINESCENCE

In Fig. 3a photoluminescence spectra of the investigated QD are shown in function of the applied magnetic field. Data is recorded for H and V polarizations under magnetic field up to 7 T in Voigt configuration. Clear four-fold line splitting can be observed, which is characteristic for the trion and following selection rules shown in Fig. 1b in the main text. Particular transition peaks are fit using Lorentzian function and derived energy shifts in respect to zero magnetic field plotted in Fig. 3b. By calculating the energy splitting between H and V polarized peaks, we recover the Zeeman splitting as shown in Fig. 3c. By performing a linear fit to the data, we recover the g-factors g_V and g_H of 2.54 and 2.86, respectively. This in turn allows us to estimate the ground-state spin g-factor to be 2.7, and the excited-state-spin g-factor to be 0.16. In Fig. 3d, we plot the diamagnetic shift vs applied magnetic field, calculated as mean energy of the split lines averaged over V and H polarization. Data are fit using quadratic function and allowed to extract a diamagnetic shift coefficient of 8.2 μeV/T^2.

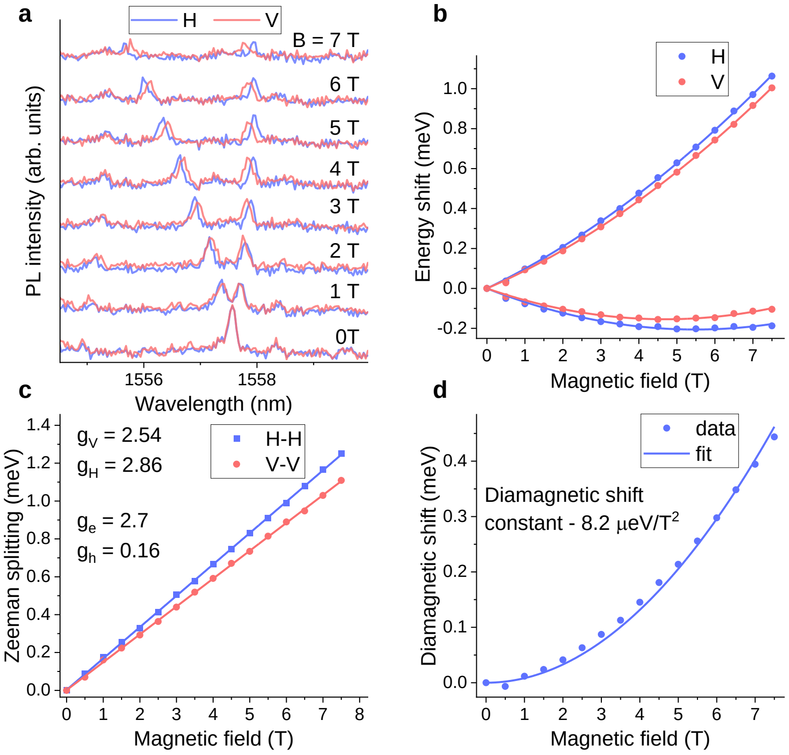

FIG. 3. **Voigt configuration magneto-photoluminescence.** (a) Photoluminescence spectra at various magnetic fields up to 7 T recorded under horizontal (H) and vertical (V) linear polarization. (b) Energy shift of the optical transitions versus applied magnetic field. A magnetic field is applied in the Voigt configuration. (c) Zeeman splitting between V and H-polarized transitions vs applied magnetic field reveling excited and ground-state spin g-factors of 0.16 and 2.7, respectively. (d) Diamagnetic shift vs applied magnetic field. By performing quadratic function fit, a diamagnetic shift constant of 8.2 μeV/T^2 was designated.

SUPPLEMENTARY NOTE 4: RATE-EQUATION MODEL

To fit the data in Fig. 3e in the main text we theoretically model the dynamics of the spin-initalization process in our system using a rate-equation model. For that, we consider four levels: two trion states $|\uparrow\Uparrow\Downarrow\rangle$, $|\downarrow\Uparrow\Downarrow\rangle$, and two ground spin states $|\Uparrow\rangle$ and $|\Downarrow\rangle$. We describe the time evolution of particular states populations $n_{|\uparrow\Uparrow\Downarrow\rangle}(t)$, $n_{|\downarrow\Uparrow\Downarrow\rangle}(t)$, $n_{|\Uparrow\rangle}(t)$ and $n_{|\Downarrow\rangle}(t)$ using following rate-equations:

$$\frac{d}{dt}n_{|\uparrow\Uparrow\Downarrow\rangle}(t) = -2n_{|\uparrow\Uparrow\Downarrow\rangle}(t)\Gamma + n_{|\Uparrow\rangle}(t)R_p + n_{|\downarrow\Uparrow\Downarrow\rangle}(t)\gamma_e - n_{|\uparrow\Uparrow\Downarrow\rangle}(t)\gamma_e, \quad (1)$$

$$\frac{d}{dt}n_{|\downarrow\Uparrow\Downarrow\rangle}(t) = -2n_{|\downarrow\Uparrow\Downarrow\rangle}(t)\Gamma + n_{|\uparrow\Uparrow\Downarrow\rangle}(t)\gamma_e - n_{|\downarrow\Uparrow\Downarrow\rangle}(t)\gamma_e, \quad (2)$$

$$\frac{d}{dt}n_{|\Uparrow\rangle}(t) = n_{|\uparrow\Uparrow\Downarrow\rangle}(t)\Gamma + n_{|\downarrow\Uparrow\Downarrow\rangle}(t)\Gamma - n_{|\Uparrow\rangle}(t)R_p + n_{|\Downarrow\rangle}(t)\gamma_h - n_{|\Uparrow\rangle}(t)\gamma_h, \quad (3)$$

$$\frac{d}{dt}n_{|\Downarrow\rangle}(t) = n_{|\uparrow\Uparrow\Downarrow\rangle}(t)\Gamma + n_{|\downarrow\Uparrow\Downarrow\rangle}(t)\Gamma + n_{|\Uparrow\rangle}(t)\gamma_h - n_{|\Downarrow\rangle}(t)\gamma_h, \quad (4)$$

where Γ is trion spontaneous recombination rate, R_p is spin pumping rate proportional to the pumping power, γ_e is trion electron spin-flip rate and γ_h is hole spin-flip rate (lifetime of the ground spin state). We solve the equations numerically assuming that after the charge injection pulse, the system is prepared in the random trion state, such that initial populations are $n_{|\uparrow\Uparrow\Downarrow\rangle}(0) = 0.5$, $n_{|\downarrow\Uparrow\Downarrow\rangle}(0) = 0.5$, $n_{|\Uparrow\rangle}(0) = 0$ and $n_{|\Downarrow\rangle}(0) = 0$. To fit the data in the Fig. 3e in the main text, we calculate the $|\Uparrow\rangle$ and $|\Downarrow\rangle$ states populations $n_{|\Uparrow\rangle}(t)$ and $n_{|\Downarrow\rangle}(t)$ at the time t equal to 4.5 ns, and plot them in function of the spin-pumping-rate for various values of spin-flip rates and fixed to $1/1.4$ ns^{-1} spontaneous emission rate (taken from a time-resolved experiments). We found that good fit to the experimental data is achieved for $1/\gamma_h$ values above 400 ns, which sets the lower limit of the hole spin lifetime in our QDs, and is also similar to previously reported values of 100 ns to 10 ms [2–4] in 800-1000 nm emitting InAs/GaAs self-assembled QDs. We also found that the trion spin-flip rate γ_e does not influence the $n_{|\Uparrow\rangle}(t)$ evolution so that we fix it to 1 ns^{-1}. In Fig. 4a we show simulated initialization fidelity after 4.5 ns spin-pumping time and in Fig. 4b time evolution of spin $|\Uparrow\rangle$ population during the optical pumping process. From graph b we can extract the theoretical spin-initialization fidelity of 96% and 99% after 4.5 ns and 6 ns optical spin-pumping time, respectively.

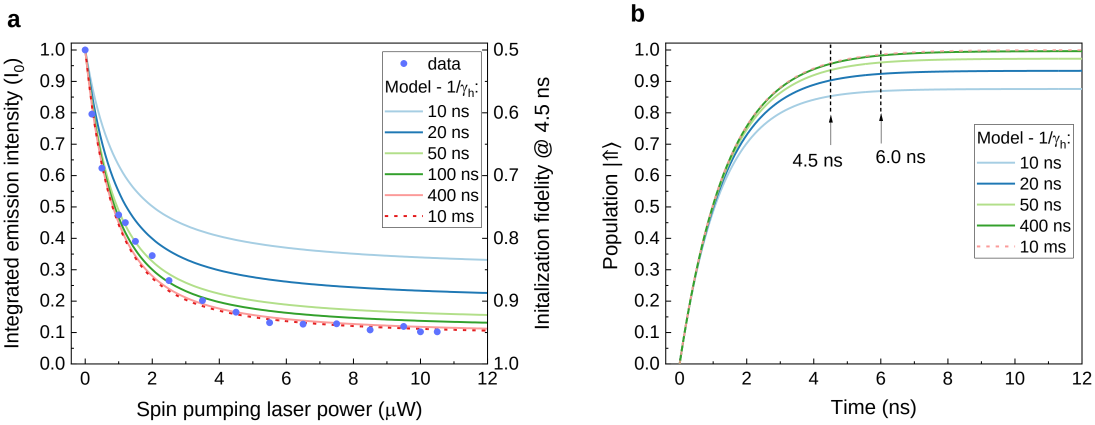

FIG. 4. **Spin initialization fidelity and dynamics.** (a) Spin initialization fidelity vs spin-pumping laser power calculated for various spin-flip rates. Simulations are performed for 4.5 ns optical pumping time. Equally good fit is obtained for $1/\gamma_h$ in the range of 400 ps to 10 ms. (b) Time evolution of spin $|\Uparrow\rangle$ population during the optical pumping process for various hole spin-flip rates γ_h. Simulations are performed for optical spin-pumping rate well above the saturation value($1/R_p = 100$ ns^{-1}).

SUPPLEMENTARY REFERENCES

* lukaszd@princeton.edu

[1] C. Carmesin, F. Olbrich, T. Mehrtens, M. Florian, S. Michael, S. Schreier, C. Nawrath, M. Paul, J. Höschele, B. Gerken, J. Kettler, S. L. Portalupi, M. Jetter, P. Michler, A. Rosenauer, and F. Jahnke, Structural and optical properties of InAs/(In)GaAs/GaAs quantum dots with single-photon emission in the telecom C-band up to 77 K, Physical Review B **98**, 125407 (2018).

[2] M. Kroutvar, Y. Ducommun, D. Heiss, M. Bichler, D. Schuh, G. Abstreiter, and J. J. Finley, Optically programmable electron spin memory using semiconductor quantum dots, Nature **432**, 81 (2004).

[3] D. Heiss, S. Schaeck, H. Huebl, M. Bichler, G. Abstreiter, J. J. Finley, D. V. Bulaev, and D. Loss, Observation of extremely slow hole spin relaxation in self-assembled quantum dots, Physical Review B **76**, 241306 (2007).

[4] B. D. Gerardot, D. Brunner, P. A. Dalgarno, P. Öhberg, S. Seidl, M. Kroner, K. Karrai, N. G. Stoltz, P. M. Petroff, and R. J. Warburton, Optical pumping of a single hole spin in a quantum dot, Nature **451**, 441 (2008).
